# Tumor Progression, Microenvironments, and Therapeutics

**DOI:** 10.3390/life12101599

**Published:** 2022-10-14

**Authors:** Hung-Chi Cheng, Li-Tzu Huang

**Affiliations:** 1 Department of Biochemistry and Molecular Biology, National Cheng Kung University, Tainan 70101, Taiwan; 2 The Institute of Basic Medical Sciences, College of Medicine, National Cheng Kung University, 1 University Road, Tainan 70101, Taiwan

Tumor malignancy starts from transformation [1,2,3], apoptotic suppression [4] tumor proliferation [5,6,7], followed by tumor progression [2,8,9,10], epithelial–mesenchymal transition (EMT), and tumor/metastasis-initiating stemness [6,11,12], with which the tumor microenvironments of primary tumor tissues are closely cross-talked [8]. Primary tumor cells then enter the bloodstream to become circulating tumor cells (CTCs) and colonize into distant organs to form secondary metastatic tumor tissues [4,13,14] that lead to cancer death [15]. Although numerous efforts, e.g., chemo-, radiation-, and target-therapies, have been made to kill tumor cells, the outcome of these therapeutic strategies often leads to distant relapse and increased patient mortality due to cancer stemness-mediated drug resistance and cancer metastasis [2,6,12,16]. Therefore, precise diagnosis [17] and alternative therapeutic strategies employing less cytotoxic drugs that may target various steps of tumor progression and tumor microenvironments [17,18] and circumvent unwanted adverse effects of drugs [14,19] are urgently needed. In this topical collection, advances in molecular mechanisms underlying tumor progression and therapeutic approaches, either targeting tumor cells or tumor microenvironments, to fight against cancer are presented. In these approaches, less cytotoxic drugs, including repurposed drugs, phytochemical agents, synthetic compounds or small molecules, and nutrients in food serving as alternative therapeutics are identified.

To accurately study molecular mechanisms underlying cancer malignancies and develop therapeutics against cancer progression, precise cancer type diagnosis and differentiation are mandatory. Stanciu et al. have presented a case report for clinical oncologists to make a better diagnosis for one type of thyroid carcinoma, carcinoma showing thymus-like differentiation (CASTLE), which is a low-grade thyroid carcinoma with indolent clinical course and favorable prognosis but otherwise it is difficult to make a precise and differential diagnosis from other more aggressive thyroid carcinomas [17]. One of the most important characteristics is that CASTLE shows positive CD5 immunoreactivity as compared to other thyroid carcinomas. Besides the methylation of lysine residues in p53, an important tumor suppressor protein, by lysine methyltransferases (KMTs), arginine residues can also be methylated by protein arginine methyltransferases (PRMTs). Since the upregulation or aberrant splicing of PRMT1 has been observed in many types of malignancies, Liu et al. have demonstrated that PRMT1 knockdown activated the p53 signal pathway where the transcriptional activity of p53 was inhibited upon direct binding and methylation of p53 by PRMT1, leading to cell growth arrest and senescence [3]. The role of binding between PRMT1 and p53 has important preclinical and clinical significance in breast cancer transformation and progression. The upper aerodigestive tract (UADT) is highly susceptible to multiple primary cancers originating from squamous epithelia and constitutes a field of cancerization. The East Asian-specific dysfunctional ALDH2*2 missense mutation is a genetic risk factor for UADT cancer. Chen et al. have reviewed the current advances to suggest alcohol consumption and *ALDH2* polymorphism as risk factors for UADT cancer progression and prognosis [2]. They have also highlighted a need for precision medicine-based preventive and therapeutic strategies by integrating lifestyle and genetic risk factors, such as genotypes of the alcohol metabolizing genes, *ADH1* and *ALDH2*, into a risk assessment model for better screening, surveillance, and treatment outcomes.

Deubiquitinase (DUB) is an essential component in the ubiquitin–proteasome system (UPS) by removing ubiquitin chains from substrates, thus modulating the expression, activity, and localization of many proteins that contribute to tumor development and progression. Li et al. have examined current advances in the effects and molecular mechanisms of DUBs in breast cancer, providing novel insight into treatments of breast cancer-targeting DUBs [9]. To this end, DUBs have emerged as promising prognostic indicators and drug targets, and specific DUB inhibitors have also been identified and are expected to benefit breast cancer patients in the future. Furthermore, dysregulated HOX transcript antisense intergenic RNA (HOTAIR) in breast cancer endogenously competes with several microRNAs and subsequently upregulates the levels of targeted downstream messenger RNAs, leading to aberrant signal transductions and further cancer progression. Shi et al. have summarized current knowledge for the induction of HOTAIR in breast cancer and its impacts on cell proliferation, migration, apoptosis, therapeutic resistance, and the underlying mechanisms, providing new thoughts for anti-cancer therapeutic strategies against breast cancer [10]. During progression, the proliferation of tumor cells is promoted and apoptosis, including detached cell death anoikis, is suppressed. Lu et al. repurposed Midazolam (MDZ), a common sedative drug acting through the γ-aminobutyric acid receptor in the central nervous system or benzodiazepine receptor (PBR) in peripheral tissues, to inhibit transforming growth factor β (TGF-β)-induced cancer cell proliferation, migration, invasion, and EMT mainly through PBR binding [6]. Their findings also unveiled a possibility that MDZ may potentially be effective in suppressing the growth of tumor-initiating cells, in that it inhibited the TGF-β-triggered Smad phosphorylation, a process that has been observed in mesenchymal stem cells, upon binding to PBR. Tsai et al. have also identified the Wnt/β-catenin pathway promoting autophagy to mediate the formation of ionizing radiation-induced glioblastoma stem-like cells and radiation resistance [12].

Alternatively, Lin et al. used active secondary metabolites, Lu01-M, from the marine *Streptomyces* sp. to inhibit the proliferative activities of several prostate cancer cell lines through multiple mechanisms including cell apoptosis, necroptosis, autophagy, ER stress, and inhibiting colony formation and cell migration [5]. Once tumor cells in primary tissues enter the bloodstream, they are immediately confronted with the loss of attachment and threat of anoikis that is often circumvented by metastatic cells as a result of biochemical and molecular transformations. Adeshankin et al. have identified deregulated glucose metabolism, oxidative phosphorylation, and proteasome in anchorage-independent cells compared to adherent cells. They used metformin, an anti-diabetic drug that reduces blood glucose (also known to inhibit mitochondrial Complex I), or proteasome inhibitors alone to increase misfolded protein accumulation, sensitized tumor cells to anoikis, and impaired pulmonary metastasis in the B16F10 melanoma model through reducing cellular ATP production and activating AMPK to foster pro-apoptotic unfolded protein response. Their findings mechanistically provide insights into anoikis resistance and identified metformin and proteasome inhibitors as potential therapeutic options for tumor metastasis [4]. In primary tumor tissues, microenvironments including innate and adaptive immunities are major hampering obstacles against which tumor cells escape and evolve to progress and intravasate into blood vessels. Moreover, before colonizing distant organs and forming metastatic lesions, the anoikis-resistant circulating tumor cells (CTCs) must possess the ability to escape immunosurveillance in the circulation and specifically attach to endothelia in the vasculature of distant organs. Rounis et al. and Trifylli et al. have both reviewed and deeply explored how immune systems impact tumor progression, distant metastasis, and cancer cachexia. They have finally come up with new and possible immunotherapeutic strategies to fight against cancer [18,20].

Finally, this collection also presents several alternative cancer-targeting vitamin-derived nutrients, natural reagents, and a oncolytic vaccinia virus. Huang et al. have exhibited evidence to suggest an inhibitory role of the biologically active form of vitamin D, calcitriol (1,25-dihydroxyvitamin D_3_), in colorectal cancer cell proliferation, angiogenesis, and disease progression [7]. Huang et al. have identified α-mangostin (α-MG), the major component in mangosteen pericarps (MP) extracts, serving as a dietary nutraceutical to therapeutically, but not prophylactically, prevent distant metastasis by suppressing periFN assembly on (suspended tumor cells) STCs within the circulation. Oral gavage with MP extracts indeed therapeutically prevents lung metastasis of STCs [14]. Vasileva et al. used a recombinant vaccinia virus, VV-GMCSF-Lact, with deletions of the viral thymidine kinase and growth factor genes and insertions of the granulocyte–macrophage colony-stimulating factor and ototoxic protein lactaptin genes to perform virotherapy in SCID mouse glioblastoma tumor growth and metastasis models. They found that VV-GMCSF-Lact significantly lowered the viability of glioblastoma cells, effectively passed the blood–brain barrier, and selectively replicated into and inhibited glioblastoma xenograft and metastasis growth [19].

Overall, the articles collected in this special topical collection partially explore current advances in understanding the molecular mechanisms underlying tumor transformation and progression and providing alternative therapeutic possibilities against cancer. We wish that this topical collection continues to attract more scientific contributions from all cancer research-related areas to further knowledge about how cancer progresses and how it can be treated.

## References

[1] Wijewardhane N., Dressler L., Ciccarelli F.D. (2021). Normal Somatic Mutations in Cancer Transformation. Cancer Cell.

[2] Chen C.-H., Wang W.-L., Hsu M.-H., Mochly-Rosen D. (2022). Alcohol Consumption, *ALDH2* Polymorphism as Risk Factors for Upper Aerodigestive Tract Cancer Progression and Prognosis. Life.

[3] Liu L.-M., Tang Q., Hu X., Zhao J.-J., Zhang Y., Ying G.-G., Zhang F. (2021). Arginine Methyltransferase PRMT1 Regulates p53 Activity in Breast Cancer. Life.

[4] Adeshakin F.O., Adeshakin A.O., Liu Z., Cheng J., Zhang P., Yan D., Zhang G., Wan X. (2022). Targeting Oxidative Phosphorylation-Proteasome Activity in Extracellular Detached Cells Promotes Anoikis and Inhibits Metastasis. Life.

[5] Lin H.-Y., Lin Y.-S., Shih S.-P., Lee S.-B., El-Shazly M., Chang K.-M., Yang Y.-C.S.H., Lee Y.-L., Lu M.-C. (2021). The Anti-Proliferative Activity of Secondary Metabolite from the Marine *Streptomyces* sp. against Prostate Cancer Cells. Life.

[6] Lu H.-L., Wu K.-C., Chen C.-W., Weng H.-K., Huang B.-M., Lin T.-Y., Liu M.-H., So E.-C., Lin R.-M., Wang Y.-K. (2021). Anticancer Effects of Midazolam on Lung and Breast Cancers by Inhibiting Cell Proliferation and Epithelial-Mesenchymal Transition. Life.

[7] Huang C.-Y., Weng Y.-T., Li P.-C., Hsieh N.-T., Li C.-I., Liu H.-S., Lee M.-F. (2021). Calcitriol Suppresses Warburg Effect and Cell Growth in Human Colorectal Cancer Cells. Life.

[8] Quail D.F., Joyce J.A. (2013). Microenvironmental regulation of tumor progression and metastasis. Nat. Med..

[9] Li S., Zhang H., Wei X. (2021). Roles and Mechanisms of Deubiquitinases (DUBs) in Breast Cancer Progression and Targeted Drug Discovery. Life.

[10] Shi Y., Huang Q., Kong X., Zhao R., Chen X., Zhai Y., Xiong L. (2021). Current Knowledge of Long Non-Coding RNA HOTAIR in Breast Cancer Progression and Its Application. Life.

[11] Pastushenko I., Blanpain C. (2019). EMT Transition States during Tumor Progression and Metastasis. Trends Cell Biol..

[12] Tsai C.-Y., Ko H.-J., Huang C.-Y., Lin C.-Y., Chiou S.-J., Su Y.-F., Lieu A.-S., Loh J.-K., Kwan A.-L., Chuang T.-H. (2021). Ionizing Radiation Induces Resistant Glioblastoma Stem-Like Cells by Promoting Autophagy via the Wnt/β-Catenin Pathway. Life.

[13] Massagué J., Obenauf A.C. (2016). Metastatic colonization by circulating tumour cells. Nature.

[14] Huang L.-T., Kuo C.-H., Tseng L., Li Y.-S., Cheng L.-H., Cheng C.-Y., Sheu S.-R., Chang W.-T., Chen C.-C., Cheng H.-C. (2022). Alpha-Mangostin Reduces Pericellular Fibronectin on Suspended Tumor Cells and Therapeutically, but Not Prophylactically, Suppresses Distant Metastasis. Life.

[15] Hanahan D. (2022). Hallmarks of Cancer: New Dimensions. Cancer Discov..

[16] Kim J.M., Jung H.A., Choi J.S., Lee N.G. (2010). Identification of anti-inflammatory target genes of *Rhizoma coptidis* extract in lipopolysaccharide-stimulated RAW264.7 murine macrophage-like cells. J. Ethnopharmacol..

[17] Stanciu M., Ristea R.P., Popescu M., Vasile C.M., Popa F.L. (2022). Thyroid Carcinoma Showing Thymus-like Differentiation (CASTLE): A Case Report. Life.

[18] Trifylli E.-M., Koustas E., Papadopoulos N., Sarantis P., Aloizos G., Damaskos C., Garmpis N., Garmpi A., Karamouzis M.V. (2022). An Insight into the Novel Immunotherapy and Targeted Therapeutic Strategies for Hepatocellular Carcinoma and Cholangiocarcinoma. Life.

[19] Vasileva N., Ageenko A., Dmitrieva M., Nushtaeva A., Mishinov S., Kochneva G., Richter V., Kuligina E. (2021). Double Recombinant Vaccinia Virus: A Candidate Drug against Human Glioblastoma. Life.

[20] Rounis K., Makrakis D., Gioulbasanis I., Ekman S., De Petris L., Mavroudis D., Agelaki S. (2022). Cancer Cachexia and Antitumor Immunity: Common Mediators and Potential Targets for New Therapies. Life.

